# Supplementary material for: Protein-truncating and rare missense variants in ATM and CHEK2 and associations with cancer in UK Biobank whole-exome sequence data
Source: J Med Genet. 2024 Aug 29;61(11):e110127. doi: 10.1136/jmg-2024-110127 (PMC11503094; doi:10.1136/jmg-2024-110127)
Supplement: online supplemental file 1 [file jmg-61-11-s001.pdf]

Protein-truncating and rare missense variants in *ATM* and *CHEK2* and associations with cancer in UK Biobank whole-exome sequence data

Toqir K. Mukhtar, Naomi Wilcox, Joe Dennis, Xin Yang, Marc Naven, Nasim Mavaddat, John R. B. Perry, Eugene J. Gardner, Douglas F. Easton

Supplementary Figures, Supplemental Tables and Supplemental Appendix

Supplemental Figures

Supplemental Figure 1: Cumulative absolute female breast cancer risks in *ATM* and *CHEK2* protein-truncating/missense variant carriers and the general female population by age[1].

Supplemental Figure 2: Cumulative absolute prostate cancer risks in *ATM* and *CHEK2* protein-truncating/missense variant carriers and the general male population by age.

Supplemental Figure 3: Cumulative absolute any cancer (other than non melanoma skin cancer) risks for female *ATM* and *CHEK2* protein-truncating/missense variant carriers and the general female population by age[2].

Supplemental Figure 4: Cumulative absolute any cancer (other than non melanoma skin cancer) risks for male *ATM* and *CHEK2* protein-truncating/missense variant carriers and the general male population by age.

[1]For breast cancer in *ATM* truncating variant carriers, where there was evidence of a decline of risk with age, HRs from the time-dependent model in Supplemental Table 1 were used. In all other cases a constant HR was assumed.

[2]For any cancer in females in ATM truncating variant carriers, where there was evidence of a decline of risk with age, HRs from the time-dependent model in Supplemental Table 1 were used. In all other cases a constant HR was assumed.

Supplemental Figure 1. Cumulative absolute female breast cancer risks in *ATM* and *CHEK2* protein-truncating/missense variant carriers and the general female population by age.

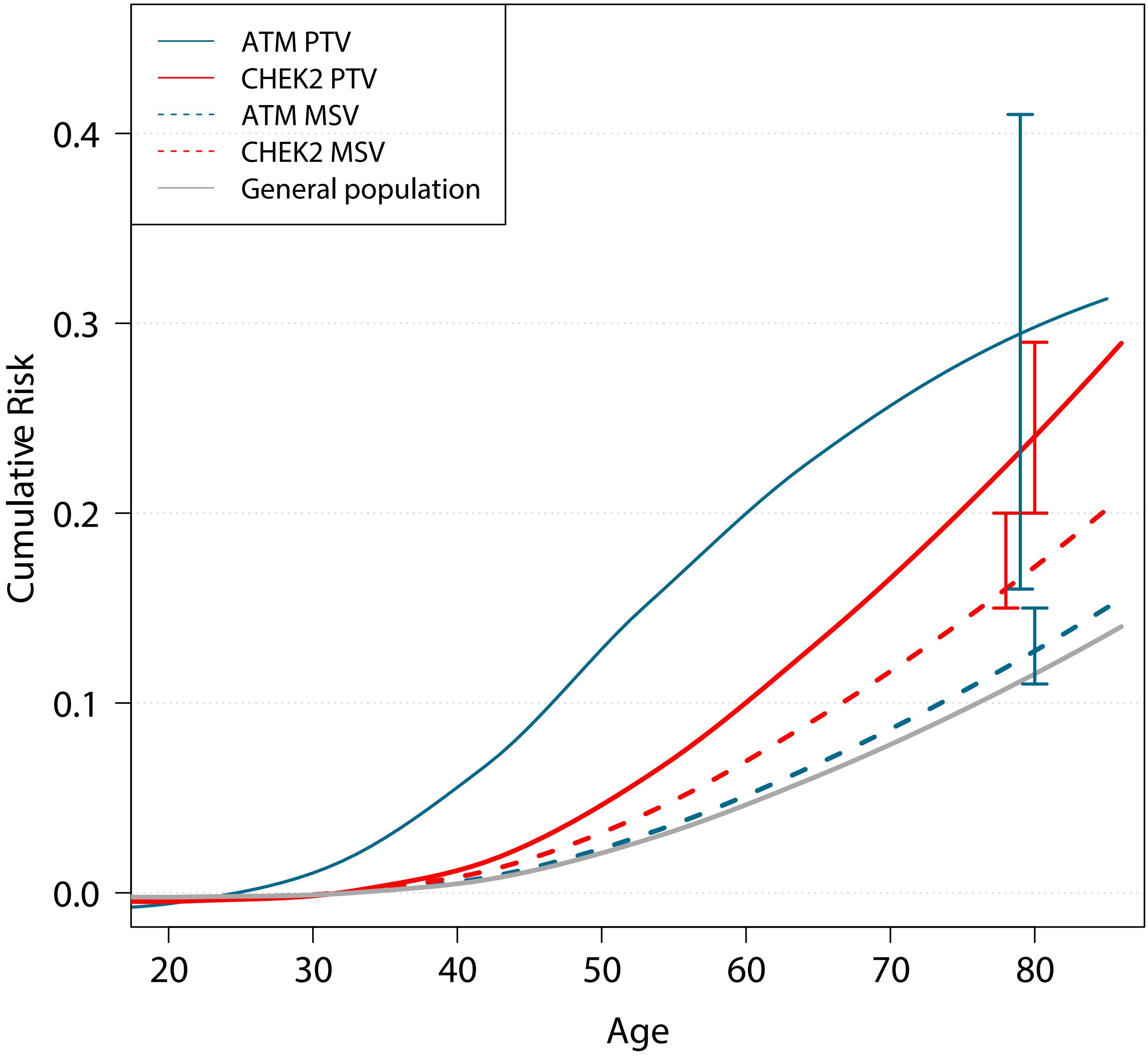

Supplemental Figure 2. Cumulative absolute prostate cancer risks in *ATM* and *CHEK2* protein-truncating/missense variant carriers and the general male population by age.

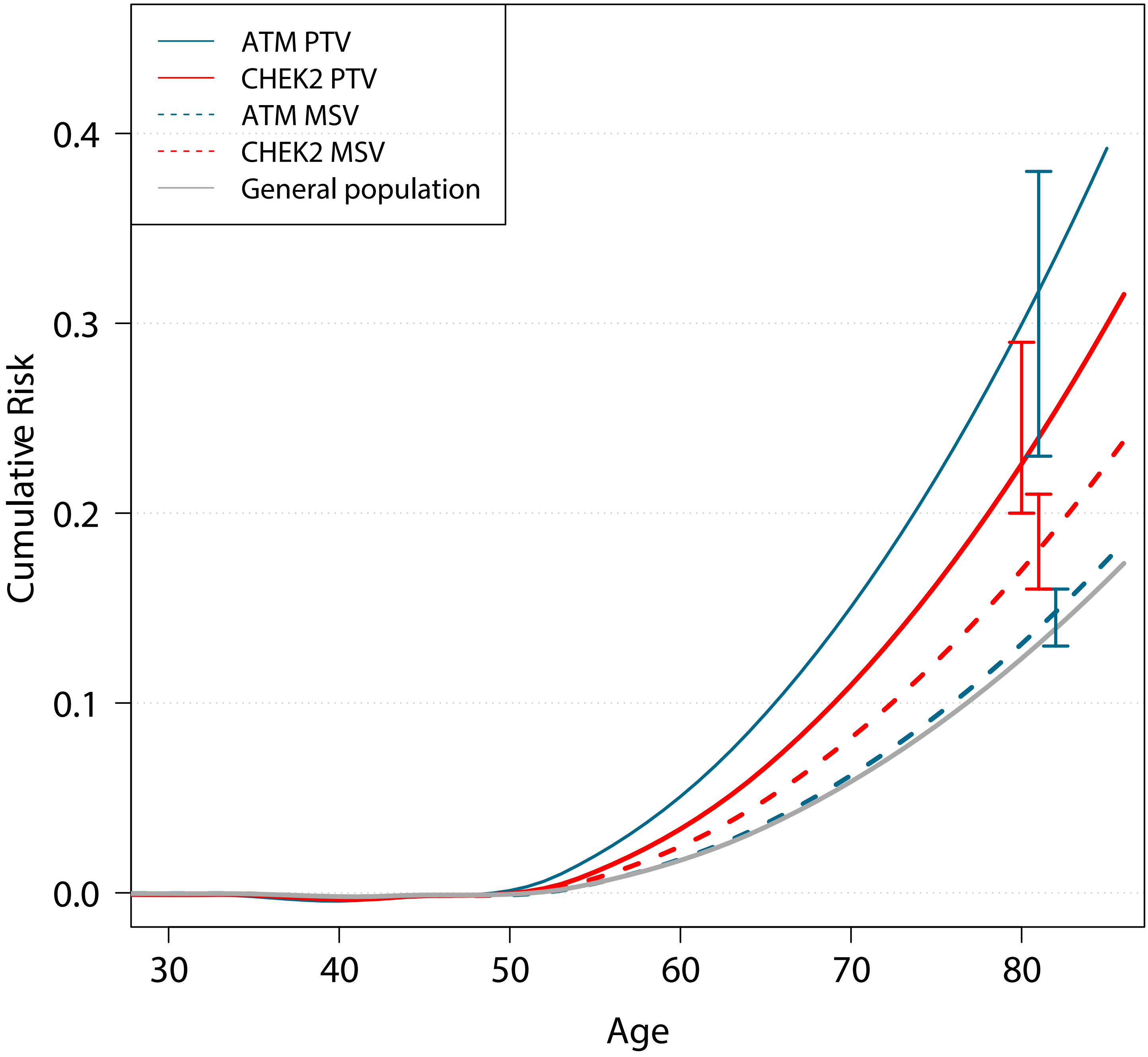

Supplemental Figure 3. Cumulative absolute risks of any cancer (other than non-melanoma skin cancer) in female *ATM* and *CHEK2* protein-truncating/missense variant carriers and the general female population by age.

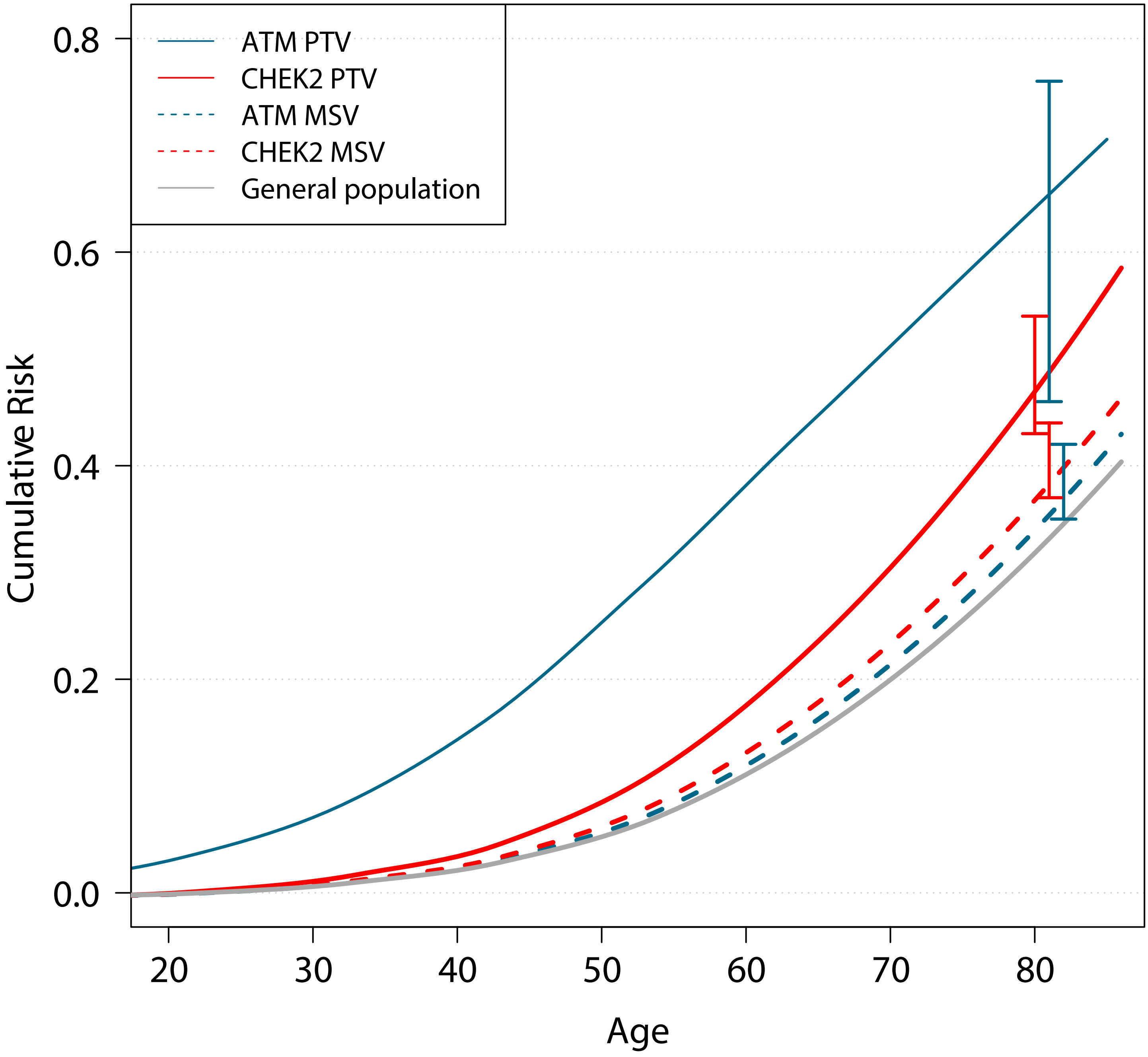

Supplemental Figure 4. Cumulative absolute risks of any cancer (other than non-melanoma skin cancer) in male *ATM* and *CHEK2* protein-truncating/missense variant carriers and the general male population by age.

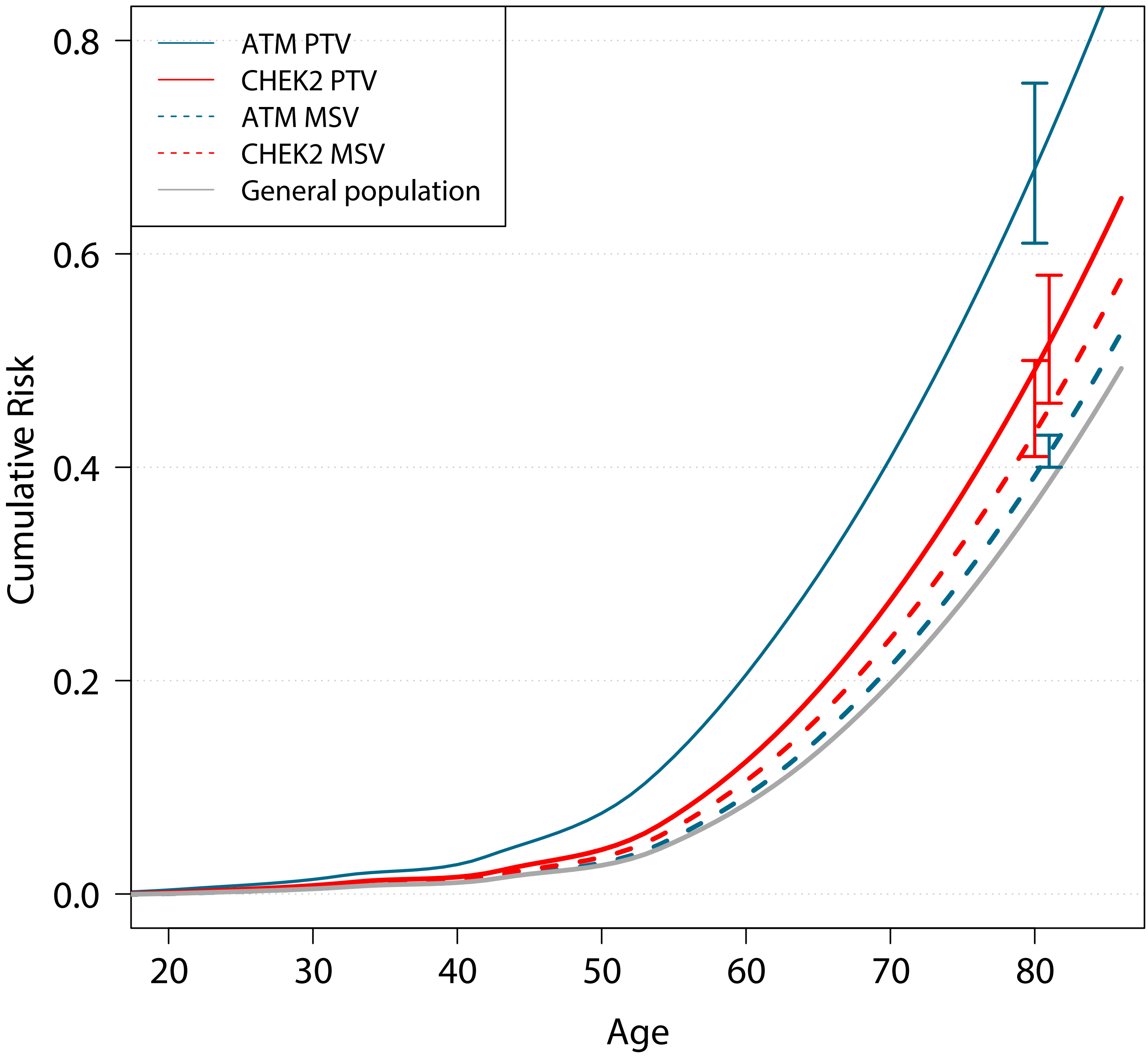

Supplemental Tables

Supplemental Table 1: Odds ratios, hazard ratios, and pooled estimates for the association of PTVs in *ATM* with cancer by type

Supplemental Table 2: Odds ratios, hazard ratios, and pooled estimates for the association of rMSVs in *ATM* with cancer by type

Supplemental Table 3: Odds ratios, hazard ratios, and pooled estimates for the association of PTVs in *CHEK2* with cancer by type

Supplemental Table 4: Odds ratios, hazard ratios, and pooled estimates for the association of rMSVs in *CHEK2* with cancer by type

Supplemental Table 5: Cox regression analyses for the association of PTVs in *ATM* with cancer by type, using a continuous time-dependent coefficient

Supplemental Table 6: Cox regression analyses for the association of rMSVs in *ATM* with cancer by type using a continuous time-dependent coefficient

Supplemental Table 7: Cox regression analyses for the association of PTVs in *CHEK2* with cancer by type, using a continuous time-dependent coefficient

Supplemental Table 8: Cox regression analyses for the association of rMSVs in *CHEK2* with cancer by type, using a continuous time-dependent coefficient

Supplemental Table 9a: Cox regression analyses for the association of PTVs in *ATM* with cancer by type using a time-dependent coefficient

Supplemental Table 9b: Cox regression analyses for the association of rMSVs in *ATM* with cancer by type using a time-dependent coefficient

Supplemental Table 10a: Cox regression analyses for the association of PTVs in *CHEK*2 with cancer by type using a time-dependent coefficient

Supplemental Table 10b: Cox regression analyses for the association of rMSVs in *CHEK2* with cancer by type using a time-dependent coefficient

Supplemental Table 11a: Odds ratios and hazard ratios for the association of rMSVs in *ATM* with pancreatic cancer, by domain and CADD score

Supplemental Table 11b: Odds ratios and hazard ratios for the association of rMSVs in *ATM* with breast cancer, by domain and CADD score

Supplemental Table 11c: Odds ratios and hazard ratios for the association of rMSVs in *ATM* with prostate cancer, by domain and CADD score

Supplemental Table 11d: Odds ratios and hazard ratios for the association of rMSVs in *ATM* with any cancer, by domain and CADD score

Supplemental Table 11e: Odds ratios and hazard ratios for the association of rMSVs in *ATM* with any cancer in females, by domain and CADD score

Supplemental Table 11f: Odds ratios and hazard ratios for the association of rMSVs in *ATM* with any cancer in males, by domain and CADD score

Supplemental Table 12a: Odds ratios and hazard ratios for the association of rMSVs in *CHEK2* with breast cancer by Helix score

Supplemental Table 12b: Odds ratios and hazard ratios for the association of rMSVs in *CHEK2* with prostate cancer by Helix score

Supplemental Table 12c: Odds ratios and hazard ratios for the association of rMSVs in *CHEK2* with pancreatic cancer by Helix score

Supplemental Table 12d: Odds ratios and hazard ratios for the association of rMSVs in *CHEK2* with any cancer in males and females by Helix score

Supplemental Table 12e: Odds ratios and hazard ratios for the association of rMSVs in *CHEK2* with any cancer in females by Helix score

Supplemental Table 12f: Odds ratios and hazard ratios for the association of rMSVs in *CHEK2* with any cancer in males by Helix score

Supplemental Table 1: Odds ratios, hazard ratios, and pooled estimates for the association of PTVs in ATM with cancer by type

**Cancer**	**ICD-**	**Carrier count**	**Non-carriers**	**OR (95% CI)**	***P*-value**	**Carrier count**	**Non-carriers**	**HR (95% CI)**	***P*-value**	**Pooled estimate (95% CI)**	***P*-value**
Oesophagus	C15	1	113	3·67 (0·51 to 26·30)	0·196	6	750	3·95 (1·77 to 8·82)	8·10[E-04]	3·90 (1·85 to 8·20)	3·43[E-04]
Stomach	C16	1	105	3·86 (0·54 to 27·69)	0·179	1	481	1·03 (0·15 to 7·36)	0·973	1·98 (0·49 to 7·97)	0·336
Colon	C18	5	1050	1·91 (0·79 to 4·60)	0·151	13	2749	2·29 (1·33 to 3·95)	2·91[E-03]	2·18 (1·37 to 3·48)	1·04[E-03]
Rectosigmoid junction	C19	2	178	4.50 (1.12 to 18.17)	0.035	1	240	2.01 (0.28 to 14.34)	0.486	3.43 (1.10 to 10.66)	0.033
Rectum	C20	4	595	2·71 (1·01 to 7·27)	0·047	1	1164	0·41 (0·06 to 2·93)	0·376	1·86 (0·78 to 4·48)	0·164
Liver and intrahepatic bile ducts	C22	0	26			2	444	2·27 (0·57 to 9·09)	0·248	2·11 (0·53 to 8·42)	
Pancreas	C25	2	39	20·64 (4·97 to 85·76)	3·10[E-05]	11	873	6·15 (3·39 to 11·14)	2·18[E-09]	7·35 (4·27 to 12·66)	6·53[E-13]
Bronchus and lung	C34	1	241	1·67 (0·23 to 11·89)	0·611	15	2715	2·71 (1·63 to 4·50)	1·21[E-04]	2·64 (1·61 to 4·32)	1·18[E-04]
Melanoma of skin	C43	9	1490	2·39 (1·24 to 4·61)	9·64[E-03]	10	2167	2·17 (1·17 to 4·04)	0·015	2·28 (1·44 to 3·59)	4·18[E-04]
Breast	C50	35	6267	2·20 (1·56 to 3·10)	7·49[E-06]	33	6169	2·34 (1·66 to 3·30)	1·09[E-06]	2·27 (1·77 to 2·91)	1·18[E-10]
Cervix uteri	C53	0	328			1	85	4·96 (0·69 to 35·65)	0·111	0·93 (0·13 to 6·63)	
Corpus uteri	C54	2	676	1·13 (0·28 to 4·54)	0·862	1	1051	0·43 (0·06 to 3·05)	0·399	0·82 (0·26 to 2·53)	0·723
Ovary	C56	4	490	3·12 (1·16 to 8·37)	0·024	5	681	3·27 (1·36 to 7·88)	8·35[E-03]	3·20 (1·66 to 6·16)	5·12[E-04]
Prostate	C61	10	2552	1·66 (0·89 to 3·12)	0·114	41	8124	2·56 (1·88 to 3·48)	1·97[E-09]	2·35 (1·78 to 3·11)	2·41[E-09]
Testis	C62	1	405	1·03 (0·15 to 7·37)	0·974	0	73			0·88 (0·12 to 6·28)	
Kidney, except renal pelvis	C64	2	386	2·11 (0·52 to 8·47)	0·294	2	1001	0·97 (0·24 to 3·86)	0·959	1·43 (0·53 to 3·82)	0·480

Bladder	C67	2	430	1·91 (0·48 to 7·68)	0·362	7	780	4·54 (2·16 to 9·55)	6·81[E-05]	3·74 (1·94 to 7·21)	8·30[E-05]
Brain	C71	1	82	4·92 (0·68 to 35·37)	0·114	1	556	0·85 (0·12 to 6·04)	0·871	2·03 (0·50 to 8·16)	0·320
Thyroid	C73	1	269	1·44 (0·20 to 10·29)	0·715	1	270	1·66 (0·23 to 11·81)	0·614	1·55 (0·39 to 6·21)	0·534
Hodgkin's disease	C81	0	183			1	81	5·88 (0·82 to 42·26)	0·078	1·59 (0·22 to 11·27)	
Diffuse non-Hodgkin's lymphoma	C83	1	297	1·36 (0·19 to 9·69)	0·760	6	790	3·68 (1·65 to 8·22)	1·47[E-03]	3·18 (1·51 to 6·69)	2·28[E-03]
Multiple myeloma and plasma cell neoplasms	C90	0	145			0	614				
Lymphoid leukaemia	C91	3	232	5·31 (1·70 to 16·62)	4·14[E-03]	4	508	3·86 (1·44 to 10·31)	7·20[E-03]	4·42 (2·11 to 9·29)	8·67[E-05]
Myeloid leukaemia	C92	0	126			0	361				
Carcinoma in situ of breast	D05	7	948	2·82 (1·33 to 5·96)	6·70[E-03]	9	1102	3·57 (1·85 to 6·88)	1·44[E-04]	3·22 (1·96 to 5·28)	4·05[E-06]
Any cancer		96	18742	2·14 (1·73 to 2·65)	2·54[E-12]	185	37617	2·34 (2·03 to 2·71)	< 2[E-16]	2·28 (2·03 to 2·56)	2·98[E-44]
Any cancer in females		61	11774	2·10 (1·60 to 2·75)	6·63[E-08]	92	17261	2·32 (1·89 to 2·85)	8·03 [E-16]	2·23 (1·88 to 2·64)	1·86[E-20]
Any cancer in females minus breast		22	4686	1·82 (1·18 to 2·79)	6.34[E-03]	50	10400	2·16 (1·64 to 2·85)	5.39[E-08]	2·06 (1·63 to 2·59)	1.03[E-09]
Any cancer in males		35	6968	2·22 (1·57 to 3·14)	7·47[E-06]	93	19996	2·37 (1·93 to 2·90)	2·00[E-16]	2·33 (1·96 to 2·77)	3·81[E-22]
Any cancer in males minus prostate		25	4416	2·47 (1·64 to 3·71)	1·32[E-05]	52	11872	2·23 (1·70 to 2·93)	7·30[E-09]	2·30 (1·83 to 2·88)	9·90[E-13]

Supplemental Table 2: Odds ratios, hazard ratios, and pooled estimates for the association of rMSVs in ATM with cancer by type

Cancer	ICD-10	Carrier count	Non-carriers	OR (95% CI)	*P*-value	Carrier count	Non-carriers	HR (95% CI)	*P*-value	Pooled (95% CI)	*P*-value
Oesophagus	C15	2	112	0·50 (0·12 to 2·03)	0·333	36	720	1·43 (1·03 to 2·00)	0·035	1·35 (0·98 to 1·87)	0·067
Stomach	C16	6	100	1·67 (0·73 to 3·82)	0·220	24	458	1·51 (1·00 to 2·27)	0·050	1·54 (1·07 to 2·23)	0·022
Colon	C18	41	1014	1·13 (0·83 to 1·54)	0·449	85	2677	0·90 (0·73 to 1·12)	0·359	0·97 (0·81 to 1·16)	0·746
Rectosigmoid junction	C19	8	172	1.28 (0.63 to 2.61)	0.492	6	235	0.73 (0.32 to 1.64)	0.445	1.00 (0.59 to 1.70)	0.995
Rectum	C20	27	572	1·32 (0·90 to 1·94)	0·162	36	1129	0·91 (0·65 to 1·26)	0·559	1·06 (0·82 to 1·37)	0·647
Liver and intrahepatic bile ducts	C22	2	24	2·35 (0·56 to 9·97)	0·245	19	427	1·27 (0·80 to 2·01)	0·313	1·35 (0·86 to 2·11)	0·190
Pancreas	C25	3	38	2·17 (0·67 to 7·03)	0·197	46	838	1·56 (1·16 to 2·10)	3·19[E-03]	1·60 (1·20 to 2·13)	1·27[E-03]
Bronchus and lung	C34	9	233	1·08 (0·56 to 2·11)	0·815	108	2622	1·18 (0·97 to 1·43)	0·092	1·18 (0·98 to 1·42)	0·090
Melanoma of skin	C43	54	1445	1·04 (0·79 to 1·36)	0·803	82	2095	1·11 (0·89 to 1·38)	0·355	1·08 (0·91 to 1·28)	0·373
Breast	C50	229	6073	1·05 (0·91 to 1·20)	0·517	236	5966	1·11 (0·97 to 1·26)	0·128	1·07 (0·97 to 1·18)	0·157
Cervix uteri	C53	12	316	1·06 (0·59 to 1·89)	0·847	2	84	0·68 (0·17 to 2·75)	0·584	1·00 (0·59 to 1·69)	0·992
Corpus uteri	C54	24	654	1·01 (0·67 to 1·52)	0·958	36	1016	0·99 (0·71 to 1·39)	0·973	1·00 (0·77 to 1·29)	0·987
Ovary	C56	8	486	0·46 (0·23 to 0·92)	0·028	27	659	1·15 (0·79 to 1·69)	0·469	0·92 (0·66 to 1·30)	0·650
Prostate	C61	118	2444	1·35 (1·12 to 1·63)	1·70[E-03]	298	7867	1·08 (0·96 to 1·21)	0·190	1·15 (1·04 to 1·27)	7·21[E-03]
Testis	C62	10	396	0·70 (0·37 to 1·31)	0·265	4	69	1·62 (0·59 to 4·43)	0·351	0·89 (0·52 to 1·51)	0·651
Kidney, except renal pelvis	C64	15	373	1·12 (0·67 to 1·87)	0·673	38	965	1·11 (0·81 to 1·54)	0·513	1·12 (0·85 to 1·48)	0·440

Bladder	C67	18	414	1·21 (0·75 to 1·93)	0·439	33	754	1·25 (0·88 to 1·77)	0·214	1·23 (0·93 to 1·64)	0·146
Brain	C71	1	82	0·34 (0·05 to 2·43)	0·282	17	540	0·89 (0·55 to 1·43)	0·619	0·84 (0·52 to 1·35)	0·470
Thyroid	C73	10	260	1·08 (0·57 to 2·03)	0·819	9	262	0·97 (0·50 to 1·88)	0·921	1·02 (0·65 to 1·62)	0·921
Hodgkin’s disease	C81	12	171	1·94 (1·08 to 3·49)	0·027	3	79	1·08 (0·34 to 3·42)	0·896	1·72 (1·02 to 2·90)	0·043
Diffuse non-Hodgkin's lymphoma	C83	12	286	1·16 (0·65 to 2·08)	0·606	29	767	1·07 (0·74 to 1·55)	0·712	1·10 (0·80 to 1·50)	0·563
Multiple myeloma and malignant plasma cell neoplasms	C90	3	142	0·58 (0·19 to 1·82)	0·351	23	591	1·11 (0·73 to 1·68)	0·632	1·03 (0·70 to 1·51)	0·896
Lymphoid leukaemia	C91	7	228	0·85 (0·40 to 1·81)	0·679	22	490	1·28 (0·83 to 1·96)	0·263	1·15 (0·79 to 1·67)	0·464
Myeloid leukaemia	C92	2	124	0·44 (0·11 to 1·79)	0·253	15	346	1·23 (0·73 to 2·06)	0·433	1·09 (0·68 to 1·77)	0·714
Carcinoma in situ of the breast	D05	37	918	1·12 (0·80 to 1·55)	0·518	45	1066	1·18 (0·87 to 1·59)	0·285	1·15 (0·92 to 1·43)	0·220
Any cancer		701	18137	1·08 (1·00 to 1·16)	0·063	1409	36393	1·10 (1·04 to 1·16)	6·41[E-04]	1·09 (1·04 to 1·14)	5·61[E-04]
Any cancer in females		417	11418	1·01 (0·91 to 1·12)	0·823	664	17049	1·09 (1·01 to 1·18)	0·024	1·06 (1·00 to 1·13)	0·060
Any cancer in females minus breast		161	4547	0·98 (0·84 to 1·15)	0·797	385	10065	1·08 (0·97 to 1·19)	0·152	1·05 (0·96 to 1·14)	0·292
Any cancer in males		284	6719	1·19 (1·05 to 1·34)	6·15[E-03]	745	19344	1·10 (1·02 to 1·19)	9·49[E-03]	1·13 (1·06 to 1·21)	2·60[E-04]
Any cancer in males minus prostate		166	4275	1·08 (0·93 to 1·27)	0·314	447	11477	1·12 (1·02 to 1·23)	0·023	1·11 (1·02 to 1·20)	0·017

Supplemental Table 3: Odds ratios, hazard ratios, and pooled estimates for the association of PTVs in CHEK2 with cancer by type

Cancer	ICD-10	Carrier count	Non-carriers	OR (95% CI)	P-value	Carrier count	Non-carriers	HR (95% CI)	P-value	Pooled estimate (95% CI)	P-value
Oesophagus	C15	2	112	3·17 (0·78 to 12·85)	0·107	7	749	1·89 (0·90 to 3·99)	0·093	2·13 (1·10 to 4·10)	0·025
Stomach	C16	0	106			3	479	1·28 (0·41 to 3·98)	0·671	1·01 (0·33 to 3·13)	
Colon	C18	9	1046	1·54 (0·80 to 2·97)	0·201	9	2753	0·65 (0·34 to 1·26)	0·203	0·99 (0·62 to 1·57)	0·957
Rectosigmoid junction	C19	3	177	3.00 (0.96 to 9.42)	0.060	0	241			1.31 (0.42 to 4.06)	
Rectum	C20	2	597	0·59 (0·15 to 2·35)	0·451	5	1160	0·86 (0·36 to 2·06)	0·731	0·77 (0·37 to 1·62)	0·491
Liver and intrahepatic bile ducts	C22	0	26			2	444	0·89 (0·22 to 3·59)	0·874	0·84 (0·21 to 3·35)	
Pancreas	C25	0	41			6	878	1·36 (0·61 to 3·04)	0·452	1·29 (0·58 to 2·87)	
Bronchus and lung	C34	0	242			14	2716	1·08 (0·64 to 1·83)	0·772	0·98 (0·58 to 1·65)	
Melanoma of skin	C43	11	1488	1·29 (0·71 to 2·35)	0·396	17	2160	1·55 (0·96 to 2·49)	0·074	1·45 (1·00 to 2·09)	0·049
Breast	C50	87	6215	2·65 (2·12 to 3·30)	<2[E-16]	67	6135	2·23 (1·76 to 2·84)	6·29[E-11]	2·44 (2·08 to 2·86)	3·61[E-28]
Cervix Uteri	C53	0	328			0	86				
Corpus uteri	C54	2	676	0·52 (0·13 to 2·08)	0·354	7	1045	1·39 (0·66 to 2·93)	0·383	1·12 (0·58 to 2·15)	0·744
Ovary	C56	6	488	2·18 (0·97 to 4·89)	0·059	8	678	2·47 (1·23 to 4·95)	0·011	2·33 (1·37 to 3·97)	1·73[E-03]
Prostate	C61	27	2535	1·83 (1·25 to 2·69)	2·09 [E-03]	84	8081	1·96 (1·58 to 2·43)	8·54[E-10]	1·92 (1·59 to 2·32)	1·18 [E-11]
Testis	C62	5	401	2·17 (0·89 to 5·25)	0·087	0	73			1·82 (0·76 to 4·38)	
Kidney, except renal pelvis	C64	5	383	2·29 (0·95 to 5·54)	0·067	8	995	1·58 (0·79 to 3·17)	0·197	1·83 (1·06 to 3·18)	0·032

Bladder	C67	3	429	1·17 (0·38 to 3·66)	0·784	5	782	1·25 (0·52 to 3·00)	0· 624	1·22 (0·61 to 2·45)	0·579
Brain	C71	2	81	4·28 (1·05 to 17·45)	0·043	0	557			0·61 (0·15 to 2·43)	
Thyroid	C73	3	267	2·07 (0·66 to 6·47)	0·211	1	270	0·76 (0·11 to 5·41)	0·783	1·61 (0·60 to 4·30)	0·343
Hodgkin's disease	C81	4	179	3·88 (1·44 to 10·48)	7·39[E-03]	2	80	5·11 (1·25 to 20·82)	0·023	4·26 (1·89 to 9·64)	4·93[E-04]
Diffuse non-Hodgkin's lymphoma	C83	4	294	2·38 (0·89 to 6·39)	0·086	7	789	1·77 (0·84 to 3·73)	0·132	1·97 (1·09 to 3·57)	0·025
Multiple myeloma and malignant cell neoplasms	C90	1	144	1·21 (0·17 to 8·62)	0·853	3	611	0·97 (0·31 to 3·02)	0·961	1·03 (0·38 to 2·74)	0·960
Lymphoid leukaemia	C91	0	235			7	505	2·73 (1·29 to 5·75)	8·49[E-03]	1·78 (0·85 to 3·74)	
Myeloid leukaemia	C92	3	123	4·07 (1·29 to 12·82)	0·017	4	357	2·17 (0·81 to 5·81)	0·125	2·81 (1·33 to 5·94)	6·75[E-03]
Carcinoma in situ of breast	D05	11	944	2·10 (1·16 to 3·82)	0·015	11	1100	2·01 (1·11 to 3·63)	0·022	2·05 (1·35 to 3·13)	8·48[E-04]
Any cancer		192	18646	1·91 (1·64 to 2·21)	<2[E-16]	297	37505	1·57 (1·40 to 1·76)	8·58[E-15]	1·68 (1·53 to 1·85)	3·44[E-27]
Any cancer in females		122	11713	1·99 (1·64 to 2·40)	1·55[E-12]	141	17572	1·67 (1·41 to 1·97)	1·59[E-09]	1·81 (1·58 to 2·06)	1·07[E-18]
Any cancer in females minus breast		26	4682	1·00 (0·67 to 1·47)	0·984	63	10387	1·28 (1.00 to 1·63)	0.054	1·18 (0.96 to 1·46)	0.124
Any cancer in males		70	6933	1·78 (1·40 to 2·28)	3·57[E-06]	156	19933	1·50 (1·28 to 1·75)	5·05[E-07]	1·57 (1·37 to 1·79)	4·21[E-11]
Any cancer in males minus prostate		43	4398	1·71 (1.26 to 2·33)	6·17[E-04]	72	11852	1·18 (0·93 to 1·48)	0·174	1·35 (1·12 to 1·62)	1·99[E-03]

Supplemental Table 4: Odds ratios, hazard ratios, and pooled estimates for the association of rMSVs in CHEK2 with cancer by type

Cancer	ICD-10	Carrier count	Non-carrier	OR (95% CI)	*P*-value	Carrier count	Non-carriers	HR (95% CI)	*P*-value	Pooled estimate (95% CI)	*P*-value
Oesophagus	C15	0	114			6	750	0·70 (0·31 to 1·56)	0·385	0·61 (0·27 to 1·35)	
Stomach	C16	0	106			3	479	0·55 (0·18 to 1·72)	0·305	0·45 (0·15 to 1·40)	
Colon	C18	15	1040	1·19 (0·71 to 1·98)	0·515	35	2727	1·11 (0·80 to 1·55)	0·529	1·14 (0·86 to 1·50)	0·369
Rectosigmoid junction	C19	1	179	0.46 (0.06 to 3.25)	0.433	2	239	0.73 (0.18 to 2.93)	0.657	0.62 (0.20 to 1.93)	0.410
Rectum	C20	10	589	1·40 (0·75 to 2·61)	0·297	11	1154	0·82 (0·45 to 1·49)	0·513	1·06 (0·58 to 1·92)	0·855
Liver and intrahepatic bile ducts	C22	0	26			5	441	1·01 (0·42 to 2·43)	0·986	0·95 (0·40 to 2·28)	
Pancreas	C25	1	40	2·03 (0·28 to 14·81)	0·484	10	874	0·99 (0·53 to 1·85)	0·979	1·06 (0·58 to 1·92)	0·855
Bronchus and lung	C34	3	239	1·05 (0·34 to 3·27)	0·939	26	2704	0·84 (0·57 to 1·24)	0·388	0·86 (0·60 to 1·25)	0·438
Melanoma of skin	C43	27	1472	1·48 (1·01 to 2·17)	0·044	30	2147	1·19 (0·83 to 1·71)	0·343	1·32 (1·01 to 1·71)	0·041
Breast	C50	112	6190	1·46 (1·20 to 1·77)	1·16[E-04]	113	6089	1·54 (1·28 to 1·85)	6·30[E-06]	1·50 (1·31 to 1·72)	1·02[E-08]
Cervix uteri	C53	5	323	1·24 (0·51 to 3·01)	0·629	0	86			1·01 (0·42 to 2·44)	
Corpus uteri	C54	8	670	0·93 (0·46 to 1·88)	0·847	14	1038	1·12 (0·66 to 1·89)	0·682	1·05 (0·69 to 1·60)	0·834
Ovary	C56	8	486	1·31 (0·65 to 2·63)	0·454	8	678	0·98 (0·49 to 1·97)	0·959	1·13 (0·69 to 1·87)	0·623
Prostate	C61	41	2521	1·37 (1·00 to 1·87)	0·051	130	8035	1·42 (1·19 to 1·69)	7·28[E-05]	1·41 (1·21 to 1·64)	1·43 [E-05]
Testis	C62	7	399	1·48 (0·70 to 3·14)	0·302	1	72	1·16 (0·16 to 8·36)	0·882	1·43 (0·71 to 2·88)	0·311
Kidney, except renal pelvis	C64	3	385	0·64 (0·21 to 1·99)	0·438	14	989	1·21 (0·71 to 2·05)	0·481	1·08 (0·67 to 1·74)	0·756

Bladder	C67	10	422	1·96 (1·05 to 3·68)	0·036	10	777	1·12 (0·60 to 2·09)	0·718	1·48 (0·95 to 2·31)	0·081
Brain	C71	0	83			4	553	0·61 (0·23 to 1·64)	0·329	0·53 (0·20 to 1·42)	
Thyroid	C73	5	265	1·52 (0·63 to 3·70)	0·351	3	268	0·93 (0·30 to 2·91)	0·906	1·27 (0·63 to 2·54)	0·507
Hodgkin’s disease	C81	3	180	1·35 (0·43 to 4·23)	0·605	1	81	1·06 (0·15 to 7·62)	0·953	1·27 (0·48 to 3·41)	0·633
Diffuse non-Hodgkin's lymphoma	C83	1	297	0·28 (0·04 to 1·96)	0·199	9	787	0·99 (0·51 to 1·91)	0·974	0·87 (0·46 to 1·63)	0·658
Multiple myeloma and malignant plasma cell neoplasms	C90	2	143	1·14 (0·28 to 4·59)	0·858	14	600	1·99 (1·17 to 3·39)	0·011	1·86 (1·13 to 3·05)	0·014
Lymphoid leukaemia	C91	5	230	1·80 (0·74 to 4·37)	0·195	14	498	2·41 (1·41 to 4·09)	1·21[E-03]	2·23 (1·42 to 3·52)	5·22[E-04]
Myeloid leukaemia	C92	0	126			8	353	1·95 (0·97 to 3·93)	0·062	1·40 (0·70 to 2·81)	
Carcinoma in situ of breast	D05	20	935	1·69 (1·09 to 2·64)	0·020	19	1092	1·42 (0·90 to 2·24)	0·127	1·55 (1·13 to 2·14)	6·82[E-03]
Any cancer		304	18534	1·35 (1·20 to 1·52)	5·01[E-07]	535	37267	1·23 (1·13 to 1·34)	2·69[E-06]	1·26 (1·18 to 1·35)	4·10[E-12]
Any cancer in females		200	11635	1·40 (1·21 to 1·62)	7·75[E-06]	253	17460	1·20 (1·06 to 1·36)	3·83[E-03]	1·26 (1·15 to 1·39)	1·09[E-06]
Any cancer in females minus breast		69	4639	1·18 (0.93 to 1·51)	0.174	122	10328	0.98 (0·82 to 1·17)	0·839	1·05 (0·91 to 1·21)	0·501
Any cancer in males		104	6899	1·28 (1·05 to 1·56)	0·016	282	19807	1·26 (1·12 to 1·42)	1·20[E-04]	1·27 (1·14 to 1·40)	4·80[E-06]
Any cancer in males minus prostate		63	4378	1·22 (0·94 to 1·56)	0·132	152	11772	1·15 (0·98 to 1·35)	0·091	1·17 (1·02 to 1·34)	0·022

Supplemental Table 5: Cox regression analyses for the association of PTVs in ATM with cancer by type, using a continuous time-dependent coefficient

	**HR (95% CI)**	***P*-value**
**Breast**		
variant	4·08 (2·49 to 6·68)	2·25[E-08]
variant x age[1]	0·95 (0·92 to 0·99)	0·012
**Prostate**		
variant	2·25 (0·82 to 6·23)	0·118
variant x age[1]	1·01 (0·96 to 1·06)	0·796
**Pancreatic**		
variant	4·30 (0·72 to 25·83)	0·110
variant x age[1]	1·02 (0·93 to 1·11)	0·672
**Any cancer females**		
variant	3·49 (2·43 to 5·01)	1·14[E-11]
variant x age[1]	0·97 (0·95 to 0·99)	0·013
**Any cancer males**		
variant	2·85 (1·64 to 4·95)	2·11[E-04]
variant x age[1]	0·99 (0·96 to 1·02)	0·487

[1]Fit using a model of the form coxph(Surv(time,event)~variant+tt(variant)+…,tt = function(x,t, ...) x*(t-50)). The main effect is thus the estimated HR associated with carrying a variant at age 50 years. The interaction term is the estimated relative change in the HR associated with carrying a variant, per year.

Supplemental Table 6: Cox regression analyses for the association of rMSVs in ATM with cancer by type using a continuous time-dependent coefficient

	**HR (95% CI)**	***P*-value**
**Breast**		
variant	1·17 (0·91 to 1·50)	0·211
variant x age[1]	1·00 (0·98 to 1·01)	0·593
**Prostate**		
variant	1·16 (0·80 to 1·68)	0·443
variant x age[1]	1·00 (0·98 to 1·02)	0·705
**Pancreatic**		
variant	1·40 (0·59 to 3·34)	0·451
variant x age[1]	1·01 (0·96 to 1·05)	0·788
**Any cancer females**		
variant	1·19 (1·01 to 1·41)	0·036
variant x age[1]	0·99 (0·99 to 1·00)	0·249
**Any cancer males**		
variant	1·06 (0·86 to 1·31)	0·602
variant x age[1]	1·00 (0·99 to 1·01)	0·688

[1]Fit using a model of the form coxph(Surv(time,event)~variant+tt(variant)+…,tt = function(x,t, ...) x*(t-50)). The main effect is thus the estimated HR associated with carrying a variant at age 50 years. The interaction term is the estimated relative change in the HR associated with carrying a variant, per year.

Supplemental Table 7: Cox regression analyses for the association of PTVs in CHEK2 with cancer by type, using a continuous time-dependent coefficient

	**HR (95% CI)**	***P*-value**
**Breast**		
variant	2·41 (1·55 to 3·74)	9·42[E-05]
variant x age[1]	1·00 (0·97 to 1·02)	0·725
**Prostate**		
variant	3·14 (1·66 to 5·95)	4·41[E-04]
variant x age[1]	0·97 (0·94 to 1·01)	0·135
**Pancreatic**		
variant	0·05 (7·90[E-04] to 2·50)	0·130
variant x age[1]	1·17 (0·99 to 1·39)	0·059
**Any cancer females**		
variant	1·93 (1·38 to 2·70)	1·12[E-04]
variant x age[1]	0·99 (0·97 to 1·01)	0·326
**Any cancer males**		
variant	1·83 (1·20 to 2·79)	0·005
variant x age[1]	0·99 (0·97 to 1·01)	0·332

[1]Fit using a model of the form coxph(Surv(time,event)~variant+tt(variant)+…,tt = function(x,t, ...) x*(t-50)). The main effect is thus the estimated HR associated with carrying a variant at age 50 years. The interaction term is the estimated relative change in the HR associated with carrying a variant, per year.

Supplemental Table 8: Cox regression analyses for the association of rMSVs in CHEK2 with cancer by type, using a continuous time-dependent coefficient

	**HR (95% CI)**	***P*-value**
**Breast**		
variant	1·72 (1·22 to 2·44)	2·21[E-03]
variant x age[1]	0·99 (0·97 to 1·01)	0·444
**Prostate**		
variant	1·57 (0·90 to 2·73)	0·113
variant x age[1]	1·00 (0·97 to 1·02)	0·716
**Pancreatic**		
variant	1·06 (0·19 to 6·11)	0·946
variant x age[1]	1·00 (0·91 to 1·09)	0·934
**Any cancer females**		
variant	1·22 (0·93 to 1·60)	0·146
variant x age[1]	1·00 (0·98 to 1·02)	0·887
**Any cancer males**		
variant	1·28 (0·92 to 1·79)	0·145
variant x age[1]	1·00 (0·98 to 1·02)	0·914

[1]Fit using a model of the form coxph(Surv(time,event)~variant+tt(variant)+…,tt = function(x,t, ...) x*(t-50)). The main effect is thus the estimated HR associated with carrying a variant at age 50 years. The interaction term is the estimated relative change in the HR associated with carrying a variant, per year

Supplemental Table 9a: Cox regression analyses for the association of PTVs in ATM with cancer by type using a time-dependent coefficient

	All cancers		Breast		Prostate		Pancreas	
**Age group**	**HR (95% CI)**	***P*-value**	**HR (95% CI)**	***P*-value**	**HR (95% CI)**	***P*-value**	**HR (95% CI)**	***P*-value**
**<50**	4·76 (2·47 to 9·19)	3·31[E-06]	3·92 (2·16 to 7·10)	6·64[E-06]	1·98 (0·28 to 14·12)	0·495	9·15 (1·26 to 66·39)	0·029
**50-59**	3·28 (2·41 to 4·48)	6·95[E-14]	3·13 (2·02 to 4·87)	3·62 [E-07]	2·56 (1·37 to 4·76)	3·06[E-03]	6·24 (2·00 to 19·50)	1·64[E-03]
**60-69**	2·04 (1·60 to 2·60)	1·05[E-08]	1·52 (0·88 to 2·62)	0·133	2·54 (1·82 to 3·54)	3·86[E-08]	4·91 (2·33 to 10·34)	2·82[E-05]
**70-79**	2·20 (1·74 to 2·78)	5·02[E-11]	1·69 (0·81 to 3·56)	0·164	2·89 (1·88 to 4·43)	1·27[E-06]	6·48 (2·90 to 14·50)	5·43[E-06]

Supplemental Table 9b: Cox regression analyses for the association of rMSVs in ATM with cancer by type using a time-dependent coefficient

	All cancers		Breast		Prostate		Pancreas	
**Age group**	**HR (95% CI)**	***P*-value**	**HR (95% CI)**	***P*-value**	**HR (95% CI)**	***P*-value**	**HR (95% CI)**	***P*-value**
**<50**	1·41 (1·01 to 1·99)	0·046	1·30 (0·97 to 1·75)	0·079	1·65 (0·94 to 2·88)	0·081	0·63 (0·09 to 4·58)	0·649
**50-59**	1·03 (0·89 to 1·19)	0·720	1·06 (0·86 to 1·30)	0·577	0·97 (0·75 to 1·25)	0·790	1·09 (0·54 to 2·21)	0·813
**60-69**	1·13 (1·04 to 1·23)	4·22[E-03]	1·05 (0·90 to 1·24)	0·528	1·08 (0·95 to 1·22)	0·246	1·62 (1·16 to 2·25)	4·57[E-03]
**70-79**	1·07 (0·99 to 1·17)	0·104	1·07 (0·84 to 1·37)	0·575	1·06 (0·90 to 1·25)	0·516	1·52 (1·01 to 2·29	0·046

Supplemental Table 10a: Cox regression analyses for the association of PTVs in CHEK2 with cancer by type using a time-dependent coefficient

	All cancers		Breast		Prostate		Pancreatic	
**Age group**	**HR (95% CI)**	***P*-value**	**HR (95% CI)**	***P*-value**	**HR (95% CI)**	***P*-value**	**HR (95% CI)**	***P*-value**
**<50**	1·69 (0·80 to 3·55)	0·170	1·97 (1·09 to 3·56)	0·026	3·07 (1·14 to 8·24)	0·026	0 (-)[a]	
**50-59**	1·61 (1·20 to 2·16)	1·71[E-03]	2·20 (1·52 to 3·19)	3·34[E-05]	2·13 (1·38 to 3·27)	5·92[E-04]	0 (-)[a]	
**60-69**	1·75 (1·48 to 2·07)	9·33[E-11]	2·47 (1·86 to 3·29)	4·59[E-10]	1·99 (1·58 to 2·51)	6·22[E-09]	1·15 (0·43 to 3·08)	0·779
**70-79**	1·39 (1·15 to 1·69)	6·91[E-04]	1·79 (1·08 to 2·98)	0·025	1·73 (1·24 to 2·40)	1·10 [E-03]	2·23 (0·92 to 5·37)	0·075

[a] no carriers

Supplemental Table 10b: Cox regression analyses for the association of rMSVs in CHEK2 with cancer by type using a time-dependent coefficient

	All cancers		Breast		Prostate		Pancreatic	
**Age group**	**HR (95% CI)**	***P*-value**	**HR (95% CI)**	***P*-value**	**HR (95% CI)**	***P*-value**	**HR (95% CI)**	***P*-value**
**<50**	1·02 (0·53 to 1·97)	0·951	1·46 (0·92 to 2·33)	0·109	0·70 (0·18 to 2·82)	0·618	1·84 (0·25 to 13·35)	0·547
**50-59**	1·33 (1·07 to 1·66)	0·011	1·83 (1·40 to 2·39)	9·41[E-06]	1·47 (1·03 to 2·10)	0·033	1·21 (0·39 to 3·79)	0·742
**60-69**	1·22 (1·06 to 1·39)	4·56[E-03]	1·53 (1·21 to 1·92)	2·90[E-04]	1·37 (1·13 to 1·66)	1·29[E-03]	0·99 (0·50 to 1·99)	0·985
**70-79**	1·22 (1·07 to 1·39)	3·49[E-03]	1·18 (0·80 to 1·74)	0·400	1·34 (1·04 to 1·74)	0·023	1·13 (0·51 to 2·53)	0·768

Supplemental Table 11a: Odds ratios and hazard ratios for the association of rMSVs in ATM with pancreatic cancer, by domain and CADD score

Domain	Carrier count	Non-carriers	OR (95% CI)	*P*-value	Carrier count	Non-carriers	HR (95% CI)	*P*-value	Pooled estimate (95% CI)	*P*-value
**Variant outside FAT and PIK**	2	39	2·02 (0·49 to 8·38)	0·332	32	852	1·53 (1·08 to 2·18)	0·018	1·56 (1·11 to 2·20)	0·011
**Variant inside FAT or PIK CADD score quintiles 1-4[1]**	1	40	3·01 (0·41 to 21·94)	0·276	10	874	1·43 (0·77 to 2·66)	0·264	1·53 (0·84 to 2·79)	0·161
**Variant inside FAT or PIK CADD score quintile 5[2]**	0	41	0		5	879	2·24 (0·93 to 5·39)	0·072	0·15 (0·06 to 0·35)	

[1]<=3.74

[2]>3.74

Supplemental Table 11b: Odds ratios and hazard ratios for the association of rMSVs in ATM with breast cancer, by domain and CADD score

Domain	Carrier count	Non-carriers	OR (95% CI)	*P*-value	Carrier count	Non-carriers	HR (95% CI)	*P*-value	Pooled estimate (95% CI)	*P*-value
**Variant outside FAT and PIK**	159	6116	1·03 (0·88 to 1·21)	0·710	155	6001	1·03 (0·88 to 1·21)	0·697	1·03 (0·92 to 1·15)	0·596
**Variant inside FAT or PIK CADD score quintiles 1-4[1]**	45	6230	0·88 (0·66 to 1·19)	0·417	62	6094	1·25 (0·98 to 1·61)	0·078	1·08 (0·89 to 1·31)	0·416
**Variant inside FAT or PIK CADD score quintile 5[2]**	30	6245	1·85 (1·28 to 2·68)	1·13[E-03]	17	6139	1·11 (0·69 to 1·78)	0·678	1·52 (1·14 to 2·04)	4·85[E-03]

[1]<=3.74

[2]>3.74

Supplemental Table 11c: Odds ratios and hazard ratios for the association of rMSVs in ATM with prostate cancer, by domain and CADD score

Domain	Carrier count	Non-carriers	OR (95% CI)	*P*-value	Carrier count	Non-carriers	HR (95% CI)	*P*-value	Pooled estimate (95% CI)	*P*-value
**Variant outside FAT and PIK**	77	2485	1·23 (0·98 to 1·55)	0·073	203	7962	1·05 (0·92 to 1·21)	0·480	1·10 (0·97 to 1·23)	0·134
**Variant inside FAT or PIK domain - CADD score quintiles 1-4[1]**	23	2539	1·14 (0·75 to 1·72)	0·550	76	8089	1·16 (0·92 to 1·45)	0·208	1·16 (0·94 to 1·42)	0·164
**Variant inside FAT or PIK domain - CADD score quintile 5[2]**	20	2542	2·99 (1·91 to 4·69)	1·89[E-06]	21	8144	0·98 (0·64 to 1·50)	0·924	1·67 (1·23 to 2·29)	1·20[E-03]

[1]<=3.74

[2]>3.74

Supplemental Table 11d: Odds ratios and hazard ratios for the association of rMSVs in ATM with any cancer, by domain and CADD score

Domain	Carrier count	Non-carriers	OR (95% CI)	*P*-value	Carrier count	Non-carriers	HR (95% CI)	*P*-value	Pooled estimate (95% CI)	*P*-value
**Variant outside FAT and PIK**	508	18330	1·11 (1·01 to 1·21)	0·030	971	36831	1·07 (1·01 to 1·14)	0·035	1·08 (1·03 to 1·14)	2·45[E-03]
**Variant inside FAT or PIK domain - CADD score quintiles 1-4[1]**	131	18707	0·86 (0·72 to 1·02)	0·089	334	37468	1·11 (1·00 to 1·23)	0·063	1·02 (0·93 to 1·13)	0·644
**Variant inside FAT or PIK domain - CADD score quintile 5[2]**	76	18762	1·56 (1·23 to 1·97)	2·16[E-04]	122	37680	1·27 (1·06 to 1·52)	8·54[E-03]	1·37 (1·19 to 1·58)	1·17[E-05]

[1]<=3.74

[2]>3.74

Supplemental Table 11e: Odds ratios and hazard ratios for the association of rMSVs in ATM with any cancer in females, by domain and CADD score

**Domain**	**Carrier count**	**Non-carriers**	**OR (95% CI)**	***P*-value**	**Carrier count**	**Non-carriers**	**HR (95% CI)**	***P*-value**	**Pooled estimate (95% CI)**	***P*-value**
**Variant outside FAT and PIK**	298	11537	1·03 (0·91 to 1·16)	0·682	451	17262	1·05 (0·96 to 1·15)	0·321	1·04 (0·97 to 1·12)	0·277
**Variant inside FAT or PIK domain - CADD score quintiles 1-4[1]**	84	11751	0·87 (0·70 to 1·09)	0·216	160	17553	1·13 (0·97 to 1·32)	0·126	1·03 (0·91 to 1·17)	0·642
**Variant inside FAT or PIK domain - CADD score quintile 5[2]**	45	11790	1·47 (1·08 to 2·00)	0·013	60	17653	1·35 (1·05 to 1·74)	0·021	1·40 (1·15 to 1·71)	8·74[E-04]

[1]<=3.74

[2]>3.74

Supplemental Table 11f: Odds ratios and hazard ratios for the association of rMSVs in ATM with any cancer in males, by domain and CADD score

**Domain**	**Carrier count**	**Non-carriers**	**OR (95% CI)**	***P*-value**	**Carrier count**	**Non-carriers**	**HR (95% CI)**	***P*-value**	**Pooled estimate (95% CI)**	***P*-value**
**Variant outside FAT and PIK**	210	6793	1·24 (1·08 to 1·43)	2·79 [E-03]	520	19569	1·10 (1·00 to 1·20)	0·041	1·13 (1·06 to 1·21)	4·43[E-04]
**Variant inside FAT or PIK domain - CADD score quintiles 1-4[1]**	47	6956	0·84 (0·63 to 1·12)	0·239	174	19915	1·08 (0·93 to 1·26)	0·290	1·02 (0·89 to 1·17)	0·751
**Variant inside FAT or PIK domain - CADD score quintile 5[2]**	31	6972	1·70 (1·18 to 2·45)	4·45[E-03]	62	20027	1·20 (0·94 to 1·54)	0·151	1·34 (1·09 to 1·65)	6·57[E-03]

[1]<=3.74

[2]>3.74

Supplemental Table 12a: Odds ratios and hazard ratios for the association of rMSVs in CHEK2 with breast cancer by Helix score

Category	Carrier count	Non-carriers	OR (95% CI)	*P*-value	Carrier count	Non-carriers	HR (95% CI)	*P*-value	Pooled estimate (95% CI)	*P*-value
**Helix score <=0·5**	61	6214	1·28 (0·99 to 1·66)	0·057	71	6085	1·56 (1·23 to 1·97)	2·11[E-04]	1·42 (1·20 to 1·69)	6·37[E-05]
**Helix score >0·5**	51	6224	1·73 (1·30 to 2·30)	1·56[E-04]	42	6114	1·50 (1·11 to 2·04)	8·35[E-03]	1·62 (1·31 to 2·01)	9·53[E-06]

Supplemental Table 12b: Odds ratios and hazard ratios for the association of rMSVs in CHEK2 with prostate cancer by Helix score

Category	Carrier count	Non-carriers	OR (95% CI)	*P*-value	Carrier count	Non-carriers	HR (95% CI)	*P*-value	Pooled estimate (95% CI)	*P*-value
**Helix score <=0·5**	25	2537	1·32 (0·88 to 1·96)	0·176	86	8079	1·47 (1·19 to 1·82)	3·70[E-04]	1·44 (1·19 to 1·74)	1·56[E-04]
**Helix score >0·5**	17	2545	1·53 (0·94 to 2·48)	0·085	44	8121	1·32 (0·98 to 1·77)	0·067	1·37 (1·07 to 1·77)	0·014

Supplemental Table 12c: Odds ratios and hazard ratios for the association of rMSVs in CHEK2 with pancreatic cancer by Helix score

Category	Carrier count	Non-carriers	OR (95% CI)	*P*-value	Carrier count	Non-carriers	HR (95% CI)	*P*-value	Pooled estimate (95% CI)	*P*-value
**Helix score <=0·5**	1	40	3·26 (0·45 to 23·76)	0·243	6	878	0·94 (0·42 to 2·10)	0·882	1·12 (0·53 to 2·36)	0·761
**Helix score >0·5**	0	41	0		4	880	1·07 (0·40 to 2·87)	0·887	0·37 (0·14 to 1·00)	

Supplemental Table 12d: Odds ratios and hazard ratios for the association of rMSVs in CHEK2 with any cancer in males and females by Helix score

Domain	Carrier count	Non-carriers	OR (95% CI)	*P*-value	Carrier count	Non-carriers	HR (95% CI)	*P*-value	Pooled estimate (95% CI)	*P*-value
**Helix score <=0·5**	174	18664	1·24 (1·06 to 1·44)	7·18[E-03]	326	37476	1·18 (1·06 to 1·32)	2·38[E-03]	1·20 (1·10 to 1·32)	1·22[E-04]
**Helix score >0·5**	131	18707	1·54 (1·29 to 1·84)	2·46[E-06]	212	37590	1·31 (1·14 to 1·50)	9·99[E-05]	1·55 (1·30 to 1·84)	1·21[E-06]

Supplemental Table 12e: Odds ratios and hazard ratios for the association of rMSVs in CHEK2 with any cancer in females by Helix score

Domain	Carrier count	Non-carriers	OR (95% CI)	*P*-value	Carrier count	Non-carriers	HR (95% CI)	*P*-value	Pooled estimate (95% CI)	*P*-value
**Helix score <=0·5**	112	11723	1·26 (1·04 to 1·53)	0·021	152	17561	1·16 (0·99 to 1·36)	0·075	1·20 (1·06 to 1·36)	3·72[E-03]
**Helix score >0·5**	88	11747	1·60 (1·28 to 1·99)	3·29[E-05]	104	17609	1·30 (1·07 to 1·57)	7·94[E-03]	1·43 (1·23 to 1·65)	1·60[E-06]

Supplemental Table 12f: Odds ratios and hazard ratios for the association of rMSVs in CHEK2 with any cancer in males by Helix score

Domain	Carrier count	Non-carriers	OR (95% CI)	*P*-value	Carrier count	Non-carriers	HR (95% CI)	*P*-value	Pooled estimate (95% CI)	*P*-value
**Helix score <=0·5**	62	6941	1·20 (0·93 to 1·55)	0·163	174	19915	1·22 (1·05 to 1·41)	9·90[E-03]	1·22 (1·06 to 1·39)	4·31[E-03]
**Helix score >0·5**	43	6960	1·44 (1·05 to 1·96)	0·022	108	19981	1·33 (1·10 to 1·60)	3·42[E-03]	1·35 (1·15 to 1·60)	3·61[E-04]

Supplemental Appendix

Variant calling, filtering, classification, and functional prediction

WES data were accessed via the UK Biobank Research Analysis Platform, enabled by DNAnexus (34). WES were processed using the OQFE mapping protocol, and variants were called using DeepVariant 0.10.0, as previously described (35). Data from individual samples were then combined into one gVCF using GLnexus 1.2.6 (36). Quality Control (QC) metrics that have been previously documented were applied to VCF files (1, 2). Variants were annotated using the Ensembl Variant Effect Predictor (VEP), version 101, using the MANE Select transcript for each gene (3). Frameshift, splice acceptor, splice donor, stop gained, and start lost variants were grouped as PTVs. PTVs in the last exon were excluded, as were those in the last 50 base positions of the penultimate exon, to exclude variants likely to avoid Nonsense-Mediated mRNA Decay (4).

Participants with unknown genetic sex, genetic sex that did not match reported sex, or sex chromosome aneuploidies, were excluded from the analyses. Participants of non-European ancestry, identified either by genetic ancestry (defined using principal components analysis) (39) or self-reported ancestry, were also excluded.

To avoid complications with inclusion of closely related individuals, one of each related pair of first-degree relatives (where the kinship coefficient >0·17) was excluded, as were participants estimated to have >10 relatives up to third-degree in the cohort.

Combined Relative Risk Estimates

Under the rare disease assumption, the OR and HR should both estimate the same underlying rate ratio or relative risk (RR). Thus, it is possible combine the estimates from the

retrospective and prospective analyses in meta-analysis. The equivalence of these estimates also depends on additional assumptions that there is no association between the variants and survival, and no differences in the RR by age. However, even if this is not exactly true, the meta-analysis should still provide a more powerful test of association.

Thus, for each gene, fixed effects meta-analyses of the ORs from the retrospective study, and HRs from the prospective study, were conducted using the log of each ratio and its standard error. Where there were zero carriers in cases in either the retrospective or prospective studies, a combined estimate was derived as (OR+OP)/(ER+EP), where OR and OP are the observed number of carriers in cases in the retrospective and prospective analysis, and ER and EP are the corresponding predicted numbers of carriers, using the predict function in R applied to the logistic or Cox regression models but without carrier status. The corresponding standard error of the log(RR) estimate is then approximately 1/sqrt(OR+OP). This method should be reasonably accurate when the (combined carrier) frequency is low, as here, since the observed counts then follow a Poisson distribution.

References

1. Gardner EJ, Kentistou KA, Stankovic S, Lockhart S, Wheeler E, Day FR, et al. Damaging missense variants in IGF1R implicate a role for IGF-1 resistance in the etiology of type 2 diabetes. Cell Genom. 2022;2(12):None.
2. Backman JD, Li AH, Marcketta A, Sun D, Mbatchou J, Kessler MD, et al. Exome sequencing and analysis of 454,787 UK Biobank participants. Nature. 2021;599(7886):628-34.
3. McLaren W, Gil L, Hunt SE, Riat HS, Ritchie GR, Thormann A, et al. The Ensembl Variant Effect Predictor. Genome Biol. 2016;17(1):122.
4. Hu C. A population-based study of genes previously implicated in breast cancer. N Engl J Med. 2021;384.
5. Lau WC, Li Y, Liu Z, Gao Y, Zhang Q, Huen MS. Structure of the human dimeric ATM kinase. Cell Cycle. 2016;15(8):1117-24.
6. Kircher M, Witten DM, Jain P, O'Roak BJ, Cooper GM, Shendure J. A general framework for estimating the relative pathogenicity of human genetic variants. Nat Genet. 2014;46(3):310-5.
7. Vroling B, Heijl S. White paper: The Helix Pathogenicity Prediction Platform. arXiv preprint 2021.
8. Dorling L, Carvalho S, Allen J, Parsons MT, Fortuno C, Gonzalez-Neira A, et al. Breast cancer risks associated with missense variants in breast cancer susceptibility genes. Genome Med. 2022;14(1):51.
